# Analysis of internet educational websites on tobacco cessation: A content analysis

**DOI:** 10.12688/f1000research.146843.1

**Published:** 2024-07-23

**Authors:** B R Avinash, Kaveri Chengappa, Ramya Shenoy, Ashwini Rao, Mithun Pai, G Rajesh

**Affiliations:** 1 Department of Public Health Dentistry, Manipal College of Dental Sciences, Mangalore, Manipal Academy of Higher Education, Manipal, Karnataka, Karnataka, Manipal, 576104, India; 2 Department of Health Outcomes and Biomedical Infodemics, Institute for child health Policy , University of Florida., Florida, Florida, USA

**Keywords:** Oral cancer, Oral health, Online resources, readability tests, Smokeless tobacco, Tobacco cessation

## Abstract

**Background:**

There have been significant changes in the lifestyles of individuals in the past few decades, which has led to increased morbidity and mortality worldwide. Both smoking and chewing forms of tobacco are highly prevalent, especially in India, and are implicated as causes of diseases, including oropharyngeal carcinomas. Effective tobacco cessation techniques and sources can help overcome addiction and reduce the disease burden in society. The aim was to evaluate the quality and readability of contents of various sources on an internet website about tobacco cessation.

**Objectives:**

i) To evaluate the readability of internet content (Google) regarding tobacco cessation using Flesch–Kincaid readability tests and the quality of internet content (Google) by using the JAMA benchmark, HONcode and DISCERN questionnaire.

**Methods:**

A content review was employed to screen the content of the Google search engine for educational tobacco cessation websites, and the top 50 websites were selected according to criteria and reviewed by two reviewers. The readability of the internet content (Google) regarding tobacco cessation was evaluated using Flesch–Kincaid readability tests. The quality of the screened sites was evaluated by using the JAMA benchmark, HONcode and DISCERN questionnaire, and the readability and quality of the screened websites were correlated using the above instruments.

**Results:**

FK readability ease was found to be 49% standard and 30% easy. The FK grade test found that 33% of the content could be easily understood by < 5 ^th^ grade. All 4 JAMA benchmarks were met by 23% of websites, and authorship was the least fulfilled criterion. Correlation analysis revealed a significant association between FK ease score and FK grade score.

**Conclusions:**

The Read-ability Ease and Read-ability Grade Levels of the websites related to tobacco cessation were not standard, and few websites fulfilled the JAMA benchmarks and had HONcode certification.

## Introduction

There have been significant changes in the lifestyles of individuals in the past few decades, which has led to increased morbidity and mortality worldwide. ^1^ Habits such as tobacco use, alcohol, diet and other lifestyle factors have been implicated as cause of diseases such as cancers of the body, including oropharyngeal cancer. ^2^^–^^4^ The disease burden is higher in underdeveloped and developing countries than in their economically stable counterparts. The hallmark of these diseases is their preventability. With effective motivation and implementation of programs aimed at tobacco control, the burden of such deadly diseases can significantly be decreased. Effective tobacco cessation techniques and sources can help overcome addiction and reduce the disease burden in society. ^5^^,^^6^

Recent decades have also witnessed a substantial rise in the number of individuals using mobile and smartphones and, with it, the internet. Everyone with a smartphone now has easy access to information on the internet, which can have a significant impact on an individual’s lifestyle. ^7^ Regardless of the background of individuals, they can now browse the internet for information related to various health-related issues and conditions. ^8^^,^^9^

Various web resources can provide information about tobacco cessation to general population. ^7^ This raises concerns about the quality of information on tobacco cessation and the contents of the same information on the internet, which may or may not be optimal, credible and standardized. There are very few investigations reported in the literature that have explored the content, quality and readability (comprehensibility) of internet resources on tobacco cessation. They can provide valuable information paving the way for standardizing this content on tobacco cessation on various websites, which are specifically tailored to reach common men.

Hence, the aim of the present study was to evaluate the quality and readability of the contents of various resources on internet websites about tobacco cessation. **Objectives:** i) To evaluate the readability of internet content (Google) regarding tobacco cessation using Flesch–Kincaid readability tests. ii) Evaluate the quality of the internet content (Google) regarding tobacco cessation by using the JAMA benchmark, HONcode and DISCERN questionnaire.

## Methods

The present study was performed using a review of the internet content on tobacco cessation. Google, a common search site, was deployed to search for content on tobacco cessation. Fifty websites that appeared first on the specific day were reviewed for their content, readability & quality. The inclusion criteria were websites that are patient focused and websites that do not involve any cost or subscription. Websites that were password protected and contained only videos and journals were excluded.

Selected websites after screening were evaluated for their quality, content and readability. Flesch–Kincaid readability tests ^10^^,^^11^ evaluated the readability of the internet source. Flesch–Kincaid readability tests include Flesch readability ease and Flesch–Kincaid grade level. The JAMA benchmark, ^12^^,^^13^ HONcode, ^13^^,^^14^ and DISCERN questionnaire were used to assess website quality. ^15^

### Ethics

Ethical approval was obtained prior to the study process from Institutional Ethics Committee. (Protocol ref no. 19051).

### Data management and statistical analysis

The parameters were entered in excel sheet and Statistical Package for Social Sciences (SPSS), version 16.0 (SPSS Inc, Chicago IL). ^16^ P value <0.05 was considered to be significant. Mean was used for descriptive data. Percentage was used to interpret FK readability tests. Mean and median was taken for DISCERN-16 questionnaire. Student’s t test was employed to evaluate HONcode Scores with DISCERN tool and FK readability scores. Pearson’s correlation was done to assess the relationship between DISCERN, FK scores and HONcode values.

## Results

Forty-three websites were reviewed. Seven websites were not included because they did not fulfil the inclusion criteria; hence A total of 21 (49%) websites were of standard Readability with an Ease Score of the Flesch–Kincaid readability test. Overall, 13 (30%) websites had a fairly easy readability ease score. Readability Grade Level analysis of the Flesch–Kincaid readability test indicates that a majority of 14 (33%) websites could be read by individuals lower than 5 ^th^ grade, while 13 (30%) websites could be read by individuals belonging to 6 ^th^ grade. A total of 5 (12%) websites could be read by individuals belonging to 8 ^th^ grade ( Table 1).

**Table 1.** Website content assessed with Flesch–Kincaid readability tests.

Flesch–Kincaid readability tests		Websites	
		n	%
Readability Ease Score	Very confusing	3	7
	Fairly difficult	4	9
	Difficult	2	5
	Standard	21	49
	Fairly easy	13	30
	Easy	0	0
Readability Grade Level	college level graduate	**0**	0
	12 ^th^ grade	**1**	2
	11 ^th^ grade	**0**	0
	10 ^th^ grade	**2**	5
	9 ^th^ grade	**1**	2
	8 ^th^ grade	**5**	12
	7 ^th^ grade	**4**	9
	6 ^th^ grade	**13**	30
	5 ^th^ grade	**3**	7
	<5 ^th^ grade	**14**	33

The findings of the current study indicate that a total of 10 (23%) of the websites satisfied all 4 JAMA benchmarks. Overall, the number of websites satisfying 1, 2 and 3 of the JAMA benchmarks was 6 (14%), 7 (16%) and 19 (44%), respectively. A total of 41 (95%) websites satisfied the JAMA benchmark of disclosure, while a total of 15 (35%) websites satisfied authorship criteria. It was also observed that a total of 8 (19%) websites did not have HONCODE certification, while a total of 35 (81%) websites had HONCODE certification.

The median and mean scores for questions in DISCERN-16. It can be observed that the mean score was maximum for item number 1 and was minimum of 2.91 for item numbers 5, 6 and 8. A majority of 21 (49%) of the websites as assessed by DISCERN-16 belonged to the fair category, while none of the websites belonged to the very poor and excellent categories.

The results of the correlation analysis indicate that there were significant associations between HonCODE certification and DISCERN total scores (p=0.021), DISCERN S1 (p=0.033) and DISCERN S2 (p=0.021). It can also be observed that there were statistically significant associations between DISCERN-S1 and DISCERN-S2, DISCERN-S1 and DISCERN-S3, DISCERN-S1 and DISCERN-16 total, DISCERN-S2 and DISCERN-S3, DISCERN-S2 and DISCERN-16 total, DISCERN-S3 and DISCERN-16 total. There were also statistically significant associations between HonCODE certification and DISCERN S1, DISCERN-S2 and DISCERN-16 total scores. Additionally, significant associations were found between FK ease and FK grade scores ( Table 2).

**Table 2.** Correlation analysis between various evaluation scores.

	DISCERN S1		DISCERN S2		DISCERN S3		DISCERN Total		FK Ease		FK Grade		HonCODE	
	r-value	p-value	r-value	p-value	r-value	p-value	r-value	p-value	r-value	p-value	r-value	p-value	r-value	p-value
DISCERN S1	-	-												
DISCERN S2	0.846 **	0.000	-	-										
DISCERN S3	0.809 **	0.000	0.789 **	0.000	-	-								
DISCERN Total	0.964 **	0.000	0.955 **	0.000	0.857 **	0.000	-	-						
FK Ease	-0.158	0.312	-0.129	0.408	-0.130	0.407	-0.150	0.336	-	-				
FK Grade	0.178	0.253	0.091	0.563	0.077	0.623	0.138	0.376	-0.935 **	0.000	-	-		
HonCODE	0.326 *	0.033	0.351 *	0.021	0.275	0.075	0.350 *	0.021	0.006	0.967	0.050	0.748	-	-

Pearsons’ correlation coefficient.

* Significant at 5 % level of significance.

** Significant at 1% level of significance.

## Discussion

There have been significant advancements in the field of information technology and mobile phone services. These developments are very dynamic and are witnessing tremendous growth every passing day. ^9^

The present study employed F–K readability, consisting of Flesch ease and grade level in place of a similar index such as the SMOG index. One of the issues with the use of the SMOG index has been its tendency to forecast readability 2 grades higher than the other estimates. The SMOG index depends upon the strict criteria of 100% accurate answers by participants. The findings of the current study indicate that the Ease Score was standard and fairly easy for the majority of the content included in the study. The current study contrasts with those reported by Wiriyakijja *et al*., ^17^ for websites on oral leukoplakia and Diniz-Freitas *et al*., ^18^ for tobacco cessation net-contents.

Material in the current study was primarily readable by individuals lower than 5 ^th^ grades and in agreement with the study conducted by Joury *et al*. ^19^ The National Institutes of Health recommended readability age is between 7 ^th^ and 8 ^th^ grades. However, reduced reading grades lead to dilution of contents.

The results of the present study indicate that 23% of the websites fulfilled all the JAMA benchmarks. In a similar study conducted by Wiriyakijja *et al*., it was noted that 17% of websites on oral leucoplakia fulfilled all four JAMA benchmarks, ^17^ and Ni Riordain *et al*., observed that 45% of websites on head and neck cancer fulfilled all four JAMA benchmarks. ^20^ Diniz-Freitas *et al*., ^18^ who observed that none of the websites related to tobacco cessation services by dentists fulfilled all 4 JAMA benchmarks. A majority of the JAMA benchmarks fulfilled in the present study include disclosure (95%) and attribution (79%). The findings are in concordance with those reported by Wiriyakijja *et al*., ^17^ for websites on oral leucoplakia but in disagreement with those observed by Diniz-Freitas *et al*., ^18^ for websites on tobacco cessation services by dentists. Authorship and currency were the frequently fulfilled JAMA benchmark in websites on adult orthodontics, as reported by McMorrow *et al*. ^21^

It can also be observed that 81% of the sites lacked HONcode certification, which is in agreement with those observed by Wiriyakijja *et al*., Diniz-Freitas *et al*.,, ^18^ and Joury *et al.* ^19^ and in disagreement with the reports of McMorrow *et al*. ^21^ It has been reported by Ni Riordain *et al*., that 39% of websites had HON code seals. ^20^ HON code assesses ethics and reputation rather than quality and information.

The number of subjects browsing internet sources for health information has considerably increased in the recent decade. ^22^ This finding sheds light on the importance of the ease and readability of available content. ^17^ Reliability of information in the field of oral cancer in different languages is also not standardized. ^23^

Reading readily available internet content pertaining to tobacco increases anxiety due to the realization of unmet health needs. ^24^ Increased levels of anxiety may also be related to poor health outcomes, and low compliance with treatment regimens and instructions. Therefore, there is a definite need for accurate information related to tobacco cessation on the internet.

Varela-Centelles *et al*., observed that the overall quality for oral cancer in health care professionals (HCP) addressed websites were of good standards. ^25^ There needs to be a consensus on what constitutes good information for patients who are seeking information on the internet on tobacco cessation.

On the other hand, it can be observed that there is information overload on the internet, and there can be information that is conflicting in nature. ^26^ The subjects need to be sensitized about the issues related to information overload, such as dubious information sources and the availability of too much information for one’s perusal. Patients may specifically be interested in the treatment outcomes associated with tobacco cessation services in particular. This may shape the patients’ expectations to be more realistic in nature, and the patients may be more receptive of various treatment outcomes. ^27^

Various factors might influence the diverse nature of information available on the internet. Various websites are fabricated by a wide variety of individuals hailing from diverse philosophical backgrounds. The purpose of website preparation may also be very different. Moreover, health literacy levels, and access to the internet may vary among patients.

Limitations of the present study will have to be borne in mind. The cross-sectional design of the study and the inclusion of a limited number of websites are its limitations. Internet content is very dynamic and is constantly changing by day. Further studies that consider a greater number of websites need to be conducted. The assessment of the content of the websites was performed by using a checklist prepared specifically for the present study. All the issues related to tobacco cessation may not be encompassed by this checklist. The websites excluded in the present study might be viewed by the patients. The present study also excluded scientific articles and books, which might be good sources of accurate information. The websites were evaluated by a dentist who was qualified and specifically trained for the purpose of the study. This type of dentist might have greater knowledge than those who check the websites. The trained dentist may also be more critical in analysing the contents of the websites than the common person.

## Conclusions

It can be observed that the read-ability ease and read-ability grade levels of the websites related to tobacco cessation were not up to the mark. Very few websites fulfilled all the JAMA benchmarks and had HONcode certification. There is a definite need for stricter rules and regulations for websites providing information related to tobacco cessation. There is a need for policy change to reinforce stricter regulation.

### Ethics and consent

Ethical clearance was obtained from the Institutional Ethics Committee of Manipal College of Dental Sciences, Mangalore (Ref No. 19051).

Informed consent: Ethical committee permission was taken and was exempted (Ref No. 19051).

## Data Availability

Figshare: Analysis of internet Educational websites on tobacco cessation health sciences. https://doi.org/10.6084/m9.figshare.24933876. ^28^ This project contains the following underlying data:
- EQIP (2).xlsx - How to quit tobacco (2).xlsx Data are available under the terms of the Creative Commons Zero “No rights reserved” data waiver (CC0 1.0 Public domain dedication).
