# Supplementary material for: Analysis of internet educational websites on tobacco cessation: A content analysis
Source: F1000Res. 2024 Nov 22;13:822. Originally published 2024 Jul 23. [Version 2] doi: 10.12688/f1000research.146843.2 (PMC11607478; doi:10.12688/f1000research.146843.2)
Supplement: Peer review history

# Reviewer report: Reviewer response for version 2

**DOI:** 10.5256/f1000research.174934.r342717

**Published:** 2024-11-29

**Authors:** Venkitachalam Ramanarayanan

**Affiliations:** 1 Department of Public Health Dentistry, Amrita School of Dentistry, Amrita Vishwa Vidyapeetham, Coimbatore, Tamil Nadu, India

No further comments

Is the work clearly and accurately presented and does it cite the current literature?

Partly

If applicable, is the statistical analysis and its interpretation appropriate?

Yes

Are all the source data underlying the results available to ensure full reproducibility?

Partly

Is the study design appropriate and is the work technically sound?

Partly

Are the conclusions drawn adequately supported by the results?

Partly

Are sufficient details of methods and analysis provided to allow replication by others?

No

Reviewer Expertise:

Dental Public Health. Evidence synthesis, Dental Education

I confirm that I have read this submission and believe that I have an appropriate level of expertise to confirm that it is of an acceptable scientific standard.

# Reviewer report: Reviewer response for version 1

**DOI:** 10.5256/f1000research.160966.r312908

**Published:** 2024-08-26

**Authors:** Tarakant Bhagat, Santosh Kumari Agrawal

**Affiliations:** 1 BP Koirala Institute of Health Sciences, Dharan, Nepal; 2 Public Health Dentistry, B. P Koirala Institute of Health Sciences, Dharan, Koshi, Nepal

**Abstract and Introduction**

 Whenever using an abbreviation, please mention it along with the full form for the first time; e.g: Flesch–Kincaid (FK), JAMA, HONcode, DISCERN etc.

**Methods**

 i. Who all were involved in the screening process? Incase multiple person were involved, how was the dispute resolved, if there was any?

 ii. Various tools were used, please mention how the scores of each tool was calculated. Also, mention the number of questions used in each tool; e.g. DISCERN-16.

**Results**

 i. What does "satisfied all 4 JAMA benchmarks" indicate? 

 ii. Please make the short-terms uniform. e.g: HONcode v HONCODE.

 iii. Table 2: Is it necessary to mention 1% significance level when in the methods section it is mentioned as 5%? Will it make any difference?

**Discussions**

 i. "Moreover, health literacy levels, and access to the internet may vary among patients" - Please provide references.

 ii. "Further studies that consider a greater number of websites need to be conducted" - What is the minimum or maximum number of websites required for content analysis?

**Conclusions**

 "It can be observed that the read-ability ease and read-ability grade levels of the websites related to tobacco cessation were not up to the mark" - but the results showed that 49% were standard content. Please justify.

Is the work clearly and accurately presented and does it cite the current literature?

Yes

If applicable, is the statistical analysis and its interpretation appropriate?

Yes

Are all the source data underlying the results available to ensure full reproducibility?

Yes

Is the study design appropriate and is the work technically sound?

Yes

Are the conclusions drawn adequately supported by the results?

Yes

Are sufficient details of methods and analysis provided to allow replication by others?

Yes

Reviewer Expertise:

Dental public health, epidemiology, oral health, health literacy

We confirm that we have read this submission and believe that we have an appropriate level of expertise to confirm that it is of an acceptable scientific standard.

# Reviewer report: Reviewer response for version 1

**DOI:** 10.5256/f1000research.160966.r306334

**Published:** 2024-08-16

**Authors:** Venkitachalam Ramanarayanan

**Affiliations:** 1 Department of Public Health Dentistry, Amrita School of Dentistry, Amrita Vishwa Vidyapeetham, Coimbatore, Tamil Nadu, India

**Introduction**

 Effective tobacco cessation techniques and sources: Kindly use a different terminology for sources. 

 Some information on the prevalence or burden of tobacco use especially among adolescents or adults could add more context to the introduction. 

 Various web resources - could add a few examples here. 

**Methods**

 There is no mention of study design. I feel it can be closely related to, though purely not, a "Health Technology Assessment"

 Google, a common search site, was deployed to search for content on tobacco cessation - What were the search words used in Google. It is very important to mention this as it determined the "hits"

 obtained. Was the wording used in tune with what a layman would use? How did the team come to a consensus on which words to use? 

 The manuscript could explain a bit more on the different scales used to assess the quality and readability ... Why were these scales chosen and what aspect it measures? .. Even though citations have been provided, it would be beneficial for the reader if a brief idea is given about these scales. 

 The parameters were entered - Parameters would be more clear if the above comment is incorporated. 

 How many investigators evaluated the websites? As some of the parameters seem subjective, How was reliability ensured?

 Would be interesting and informative to note how readability score levels were assessed. 

 The findings of the current study indicate that a total of 10 (23%) of the websites satisfied all 4 JAMA benchmarks. Overall, the number of websites satisfying 1, 2 and 3 of the JAMA benchmarks was 6 (14%), 7 (16%) and 19 (44%), respectively.  - 1, 2 and 3 of JAMA benchmarks need to be named. There is no table for this which would help the uninitiated reader to understand the tool. 

 The median and mean scores for questions in DISCERN-16. It can be observed that the mean score was maximum for item number 1 and was minimum of 2.91 for item numbers 5, 6 and 8. A majority of 21 (49%) of the websites as assessed by DISCERN-16 belonged to the fair category, while none of the websites belonged to the very poor and excellent categories. - Need a table for this. Item numbers 5,6 and 8 needs to be elaborated. 

**Discussion**

 Add more information on HONcode certification. How will a layman be able to identify it ?

 The assessment of the content of the websites was performed by using a checklist prepared specifically for the present study. - Provide checklist as an attachment 

 Would have been more interesting to explain more on the websites which were selected. Was it government websites? professional bodies? or blogs etc, ... who were the authors .. what was the content .. all of this in brief at least .. 

 What guidance do you offer to a layman to ensure that the website he reads is of good quality. Any red-flags you can propose based on your analysis?

**Conclusion**

 were not up to the mark.- Reframe using scienfic language

**Overall comments**

 - The study is interesting and relevant. But the information provided is too short, superficial and generic. There is scope for explanation in all sections of the manuscript which would help the reader follow the article better. The quantum of work done in evaluating all 41 websites using various scales is not reflecting in the manuscript.

Is the work clearly and accurately presented and does it cite the current literature?

Partly

If applicable, is the statistical analysis and its interpretation appropriate?

Yes

Are all the source data underlying the results available to ensure full reproducibility?

Partly

Is the study design appropriate and is the work technically sound?

Partly

Are the conclusions drawn adequately supported by the results?

Partly

Are sufficient details of methods and analysis provided to allow replication by others?

No

Reviewer Expertise:

Dental Public Health. Evidence synthesis, Dental Education

I confirm that I have read this submission and believe that I have an appropriate level of expertise to confirm that it is of an acceptable scientific standard, however I have significant reservations, as outlined above.
